# Glucose metabolism following human traumatic brain injury: methods of assessment and pathophysiological findings

**DOI:** 10.1007/s11011-014-9628-y

**Published:** 2014-11-21

**Authors:** Ibrahim Jalloh, Keri L. H. Carpenter, Adel Helmy, T. Adrian Carpenter, David K. Menon, Peter J. Hutchinson

**Affiliations:** 1 Division of Neurosurgery, Department of Clinical Neurosciences, University of Cambridge, Box 167 Cambridge Biomedical Campus, Cambridge, CB2 0QQ UK; 2 Wolfson Brain Imaging Centre, Department of Clinical Neurosciences, University of Cambridge, Box 65, Cambridge Biomedical Campus, Cambridge, CB2 0QQ UK; 3 Division of Anaesthesia, Department of Medicine, University of Cambridge, Box 93, Cambridge Biomedical Campus, Cambridge, Cambridge CB2 0QQ UK

**Keywords:** ^13^C labelling, Glucose, Magnetic resonance spectroscopy, Microdialysis, Positron emission tomography, Traumatic brain injury

## Abstract

The pathophysiology of traumatic brain (TBI) injury involves changes to glucose uptake into the brain and its subsequent metabolism. We review the methods used to study cerebral glucose metabolism with a focus on those used in clinical TBI studies. Arterio-venous measurements provide a global measure of glucose uptake into the brain. Microdialysis allows the in vivo sampling of brain extracellular fluid and is well suited to the longitudinal assessment of metabolism after TBI in the clinical setting. A recent novel development is the use of microdialysis to deliver glucose and other energy substrates labelled with carbon-13, which allows the metabolism of glucose and other substrates to be tracked. Positron emission tomography and magnetic resonance spectroscopy allow regional differences in metabolism to be assessed. We summarise the data published from these techniques and review their potential uses in the clinical setting.

## Introduction

Traumatic brain injury (TBI) is heterogeneous disorder caused by the action of external mechanical forces on the brain. It is one of the leading causes of mortality in young adults with mortality rates in the USA and Europe ranging between 15 and 20 per 100,000 per annum (Tagliaferri et al. 2006; Maas et al. 2008). TBI incidence is increasing worldwide. In developing countries this is due to increasing use of motorised vehicles, while in developed nations there is an increasing incidence of TBI in the older population due to falls (Roozenbeek et al. 2013). Moreover, it is estimated that approximately 5.3 and 7.7 million people are living with disability due to TBI in the USA and Europe respectively (Roozenbeek et al. 2013). TBI thus has huge economic and societal costs.

The primary injurious event is followed in the hours and days after injury by secondary pathological processes that can exacerbate damage, and are thus targets for therapy. The management of TBI centres on the maintenance of adequate cerebral blood flow (CBF) and the delivery of sufficient glucose and oxygen to preserve tissue not irreparably damaged by the initial physical insult. Thus, cerebral perfusion is maintained, hypoxia avoided, and with modern neurocritical care management, secondary ischaemia is not thought to play a major role. However, despite progress in management of TBI patients, many still suffer long-term or lifelong disabilities, thus placing heavy demands on carers and healthcare resources, and there is scope for better treatments (Kolias et al. 2013).

Increasingly recognised are the cellular perturbations that challenge energy metabolism in TBI. The following changes have been revealed by experimental studies. Disruption to cell membranes and a failure to maintain normal ionic homeostasis results in cytotoxic oedema, which further challenges membrane integrity (Unterberg et al. 2004). Excitotoxic release of glutamate and other excitatory neurotransmitters exacerbate the disturbance of trans-membrane ionic gradients (Katayama et al. 1990; Kawamata et al. 1992). Mitochondrial calcium accumulation induces oxidative stress and impairs mitochondrial function (Xiong et al. 1997; Osteen et al. 2004). The generation of free radicals, highly reactive oxygen and nitrogen species, normally kept in check by cellular antioxidant systems, leads to the oxidative modification of proteins, lipids and DNA (Hall et al. 1993; Marklund et al. 2001). This results in increased permeability of the inner mitochondrial membrane, release of cytochrome c leading to caspase-dependent apoptosis, and activation of poly-ADP ribose polymerase (PARP), a nuclear DNA repair enzyme that consumes NAD^+^_,_ a coenzyme essential for glycolysis (Sullivan et al. 1998; Lewén et al. 2001; Satchell et al. 2003). Ultimately, although initially potentially reversible, these metabolic and mitochondrial perturbations, result in energy failure and activation of both apoptotic and necrotic pathways (Lewén et al. 2001). These and other, as yet uncharacterised changes may be important in TBI patients’ brains. The pathophysiology of energy failure after TBI is not yet fully understood but has important implications for how TBI patients are managed. Improving our understanding of these metabolic disturbances will help lead to strategies that reduce secondary injury and improve outcomes.

The metabolic perturbations that result from TBI have largely been studied using animal models of TBI (Andersen and Marmarou 1992; Xiong et al. 2013). In general, for the purposes of metabolic studies, metabolites are measured and/or tracers are used to follow biochemical processes at set time points after experimental injury. Many of the techniques used require the brain to be extracted and so are not applicable in clinical studies. Animal models of TBI produce a relatively homogenous injury in comparison to clinical TBI and the majority of studies do not consider co-morbidities or other systemic factors that frequently complicate clinical TBI. Patients with severe TBI commonly have injuries to other organs, such as chest and abdominal injuries, with consequent risk of systemic hypoxia and/or hypotension, which is deleterious to outcome (Chesnut et al. 1993; Stocchetti et al. 1996). The impact of sedative drugs and other pharmaceutical agents, which also likely influence cerebral metabolism, are also frequently overlooked. Hence, we have to be careful in applying what we learn about glucose metabolism after TBI in the laboratory to what actually happens in clinical practice. For an in-depth review of experimental TBI models, see (Xiong et al. 2013).

**Fig. 1:**
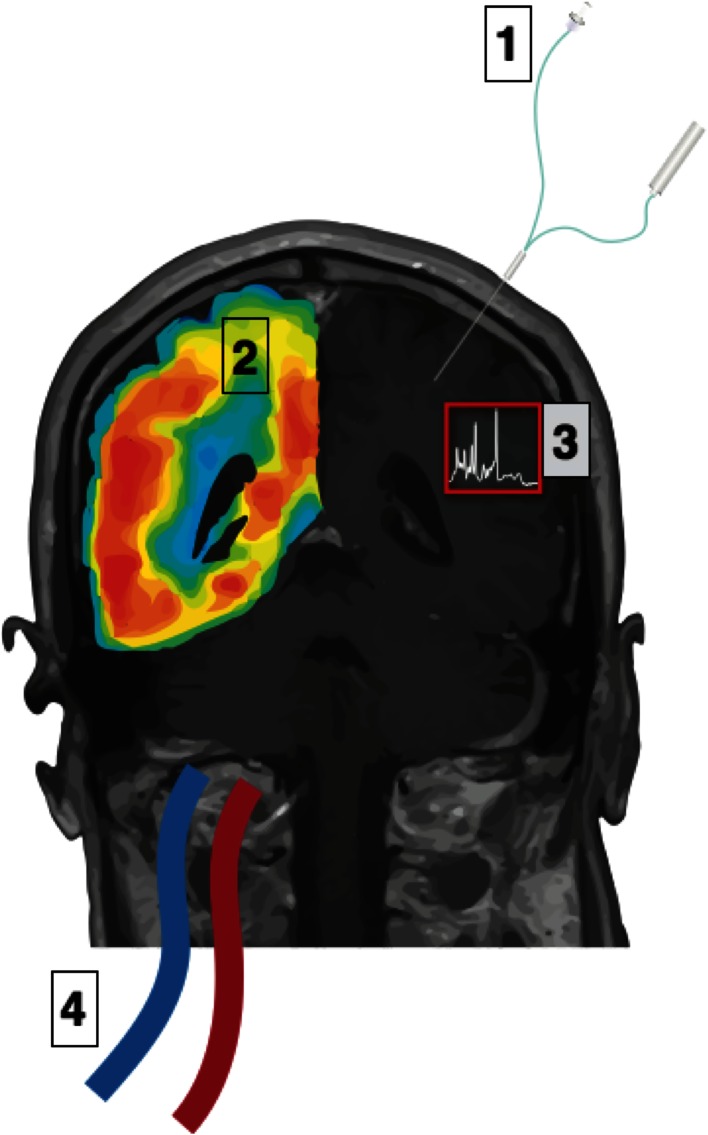
Methods for measuring glucose metabolism in the human brain: Various techniques lend themselves to measuring glucose metabolism in the human brain. (*1*) Microdialysis, which allows metabolites in the extracellular fluid to be measured, is an invasive technique requiring insertion of a microdialysis catheter (possessing a semipermeable membrane, with nominal molecular weight cut-off typically 20 kDa or 100 kDa) into brain parenchyma. A pump is used to infuse perfusion fluid at a low flow rate (e.g. 0.3 μl/min) into the catheter and returned microdialysate is collected into small vials for subsequent analysis. (*2*) Imaging techniques include positron emission tomography (PET), which uses detectors to measure ionising radiation emitted from the glucose analogue FDG administered intravenously. This provides a measure of the uptake and phosphorylation of glucose. (*3*) ^1^H and ^31^P magnetic resonance spectroscopy (MRS) can be used to measure lactate and high-energy phosphate compounds respectively, in different regions of the brain. (*4*) A global measure of uptake or release from the brain can be achieved by using a jugular bulb catheter, which allows the venous outflow of the brain to be sampled. Measured concentrations in blood sampled from the venous catheter can be compared with arterial concentrations of metabolites, typically measured in blood taken from an arterial line. This enables the net uptake or release of metabolites by the brain to be calculated and, if cerebral blood flow is known, the cerebral metabolic rate to be calculated

There are various methods for investigating metabolism after TBI in patients (Fig. 1). Measuring arterial, jugular and/or cerebrospinal fluid (CSF) concentrations of metabolites provides a measure of how much the brain as a whole imports or exports. Microdialysis, which samples extracellular fluid, is a focal technique that allows the immediate microenvironment of the brain to be sampled in vivo. Microdialysis can also be used to deliver substances and in this way has been used to deliver glucose and other metabolites labelled with carbon-13 (^13^C). This is combined with ex vivo nuclear magnetic resonance (NMR) spectroscopy of the microdialysis-collected extracellular fluid yielding information on different intracellular pathways. Autoradiography experiments in animals, and analogous positron emission tomography (PET) studies in humans, use glucose labelled with a radioactive tracer. These experiments allow regional differences in glucose uptake and metabolism to be measured. In vivo magnetic resonance spectroscopy (MRS) has also been used to measure the combined extra- and intracellular regional concentrations of lactate and high-energy phosphate compounds. Our ability to study cerebral metabolism using these techniques has greatly improved our understanding of the pathophysiology of TBI. Importantly, there is also the potential for these techniques to help in the management of patients with TBI and improve clinical outcomes although their role in the clinical setting is still being defined.

**Fig. 2:**
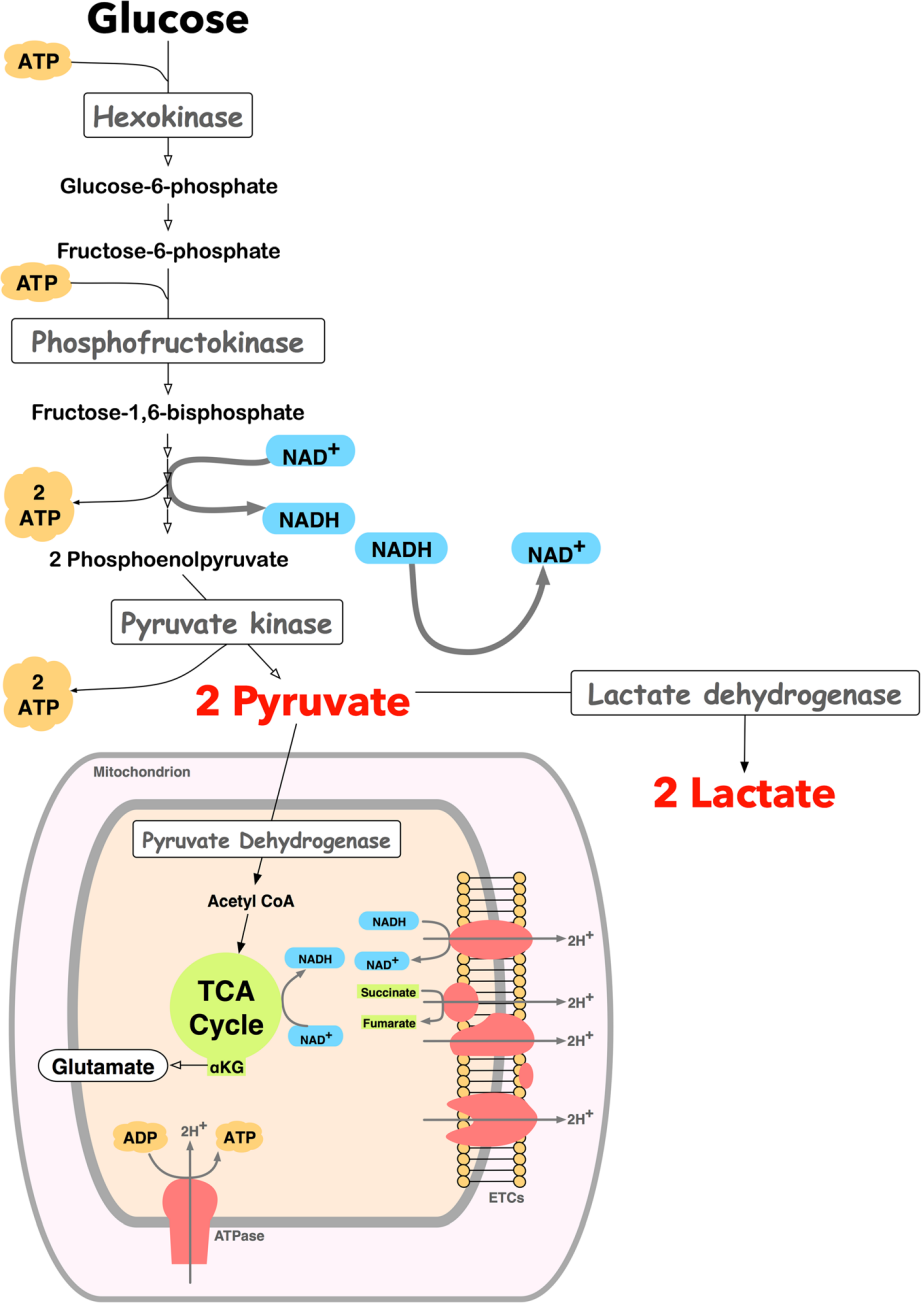
Glycolysis and the tricarboxylic acid cycle: Glucose is the preferred substrate for the brain, although the brain can take up lactate, other monocarboxylic acids and ketone bodies under certain circumstances, for example, during the perinatal period. Once it enters cells, glucose is metabolised through glycolysis to pyruvate. Glycolysis (also termed Embden-Meyerhof pathway) is the series of reactions that results in the breakdown of glucose, generating pyruvate, adenosine triphosphate (ATP) and nicotinamide adenine dinucleotide (NADH). It is a truly fundamental pathway, found throughout nature and proceeds without the need for oxygen. The ten key enzymatic steps, in which 2 ATP molecules are consumed early on but then paid back later with the generation of 4 ATP molecules per glucose molecule, so the net production of ATP is 2 molecules per molecule of glucose, takes place in the cytoplasm. There are three key regulatory points catalysed by the enzymes hexokinase, phosphofructokinase, and pyruvate kinase. These reactions are essentially irreversible whereas the other enzymatic steps exist in equilibrium. Pyruvate is converted to acetyl CoA, which enters the tricarboxylic acid (TCA) cycle, within mitochondria. The TCA cycle results in the transfer of electrons (from NADH and succinate), to electron transport chains (ETC.) located in the inner mitochondrial membrane, which ultimately deposit on oxygen molecules. Thus the TCA cycle generates carbon dioxide (also generated by the pyruvate dehydrogenase step prior to TCA cycle) and the ETC generates water. The ETCs pump protons across the inner mitochondrial membrane, maintaining a gradient of protons across the membrane. Protons then flow down their concentration gradient, through ATP synthetases (ATPase), resulting in the generation of ATP, the cells’ widely used energy currency. Energy production from glucose is intrinsically related to neurotransmission. Glutamate spins off the TCA cycle from α-ketoglutarate (αKG), an intermediate of the TCA cycle. Glutamate can be converted reversibly into glutamine. Glutamate can also be converted into gamma-aminobutyric acid (GABA). There is a constant cycle of glutamate released during neurotransmission, retrieved from synaptic junctions by astrocytes and returned to neurons as glutamine

The aim of this review is to summarise what we know about the metabolic perturbations that characterise TBI with a focus on clinical studies. Specifically, we will focus on changes to glucose metabolism after moderate to severe TBI. See Fig. 2 for an overview of glucose metabolism. The most noticeable feature is that the injured brain can exhibit a relative rise in non-oxygen-consuming glucose metabolism. With the increasing clinical availability of techniques that provide insight into glucose metabolism, such as microdialysis and MRS it is important to review the available evidence with a view to improving our ability to interpret this data.

## Arterio-venous gradient studies

Arterio-venous (AV) studies provided the first means for studying human cerebral energy metabolism. This was first achieved when Kety and Schmidt (1948) applied the Fick principle to calculate CBF and cerebral oxygen consumption. The amount of substance that a tissue removes from or releases into the circulation is equal to the product of the blood flow to the tissue and the arterio-venous (AV) concentration difference. Using this principle, the steady-state rate of utilisation or production, known as the cerebral metabolic rate (CMR), can be calculated.

AV studies investigating the CMR of glucose (CMRglc) after TBI demonstrate a depression in CMRglc indicating reduced glucose uptake and utilisation by the injured brain (Cruz 1995; Leegsma-Vogt et al. 2001; Glenn et al. 2003). Glenn et al. (2003) compared 49 TBI patients with 31 awake volunteers and used AV difference measurements and a Xenon-133 clearance technique to calculate CBF. A mean CMRglc of 4.46 ± 1.16 mg/100 g/min was found in normal subjects whereas this was significantly lower in TBI patients who had a mean CMRglc of 3.43 ± 2.32 mg/100 g/min during post-injury days 0 to 5.

Alongside this global depression in glucose metabolism, Glenn et al. (2003) found a significant depression in oxygen metabolism. They calculated the metabolic ratio (MR) by calculating CMRO_2_/CMRglc (in molar units) and found that this was significantly lower in TBI patients compared to normal subjects. The MR in normal subjects was 5.83 ± 1.41, which demonstrates the efficiency of the brains aerobic metabolism of glucose. Six moles of oxygen are needed for the oxidation of one mole of glucose hence a MR value close to 6 indicates almost complete aerobic metabolism of glucose. A MR value greater than 6 implies aerobic metabolism of other substrates, and less than 6 the anaerobic metabolism of glucose. Other substrates potentially metabolised by the injured brain that would in theory account for an MR greater than 6 include lactate (see below) and ketone bodies (Prins and Giza 2006). Further non-glucose substrates for brain could include fatty acids, although a recent review has argued against fatty acids being a good brain energy source (Schönfeld and Reiser 2013). In the TBI patients the MR was 4.11 ± 2.11, i.e. more moles of glucose metabolised per mole of oxygen, which indicates that there is proportionally greater “anaerobic” (non-oxygen-consuming) metabolism of glucose. In several patients a MR of less than 3.44 was observed and this was interpreted as indicating ‘relative hyperglycolysis’; proportionally much greater anaerobic glucose metabolism than aerobic, although only a small proportion of samples (5 %) demonstrated absolute hyperglycolysis with an elevated CMRglc. In a similar metabolic ratio calculation based on AV difference of oxygen and glucose measurements in 69 TBI patients, which they termed the oxygen-glucose index (OGI), Holbein et al. (2009) found values mostly above 6 in 69 TBI patients suggesting maintained aerobic metabolism of glucose. However, higher arterial glucose levels were associated with greater “anaerobic” (non-oxygen-consuming) glucose metabolism.

As well as perturbations to the relationship between cerebral glucose and oxygen metabolism, the metabolism of lactate is also affected by TBI. AV measurements in healthy controls reveal a small net release of lactate from the brain into the circulation (Glenn et al. 2003; Larsen et al. 2008). However, several authors have revealed periods during which AV measurements indicate uptake of lactate by the injured brain after TBI (Glenn et al. 2003; Meierhans et al. 2012; Jalloh et al. 2013). Jalloh et al. (2013) examined AV gradients in 19 patients with diffuse TBI and found periods of lactate uptake in 17 of these patients. This was associated with maintenance of normal AV glucose measurements. The likelihood is that, after TBI, lactate from the circulation is taken up by the injured brain and used as a metabolic substrate. Supporting this concept, intravenous administration of ^14^C-lactate to a rodent TBI model demonstrated accumulation of radiolabel at the injury site (Chen et al. 2000). Moreover, in humans, lactate labelled with ^13^C and delivered to the brains of TBI patients by microdialysis is metabolised via the TCA cycle to glutamine (Gallagher et al. 2009).

AV studies provide a measure of the uptake or release of substance by the whole brain at a single time point; they lack the spatial and temporal resolution to explore how changes to glucose metabolism relate to other pathophysiological changes. Moreover, given the heterogeneity of TBI this technique will not detect metabolic differences between injured and non-injured areas of brain. Another important consideration when using this method is that jugular venous samples are not representative of the entire brain; only one vein is sampled excluding contralateral and extra-jugular venous drainage (Ferris and Engel 1946). These limitations and the difficulty of obtaining reliable venous oxygen saturation measurements means that they are not used routinely in clinical practice (Pattinson et al. 2005).

## Microdialysis

Microdialysis allows continuous in vivo sampling of molecules from the extracellular fluid from the human brain and permits the longitudinal analysis of certain energy-related small molecules. Clinical microdialysis catheters are often inserted alongside brain tissue oxygen sensors such that changes in concentrations of small molecules related to energy metabolism can be monitored in conjunction with variations in tissue oxygen levels. Metabolic features of microdialysates statistically associated with adverse outcomes after TBI include high concentrations of lactate and high lactate/pyruvate ratio, while for glucose the situation appears more complex with both high and low concentrations having adverse associations. Further details are as follows.

**Fig. 3:**
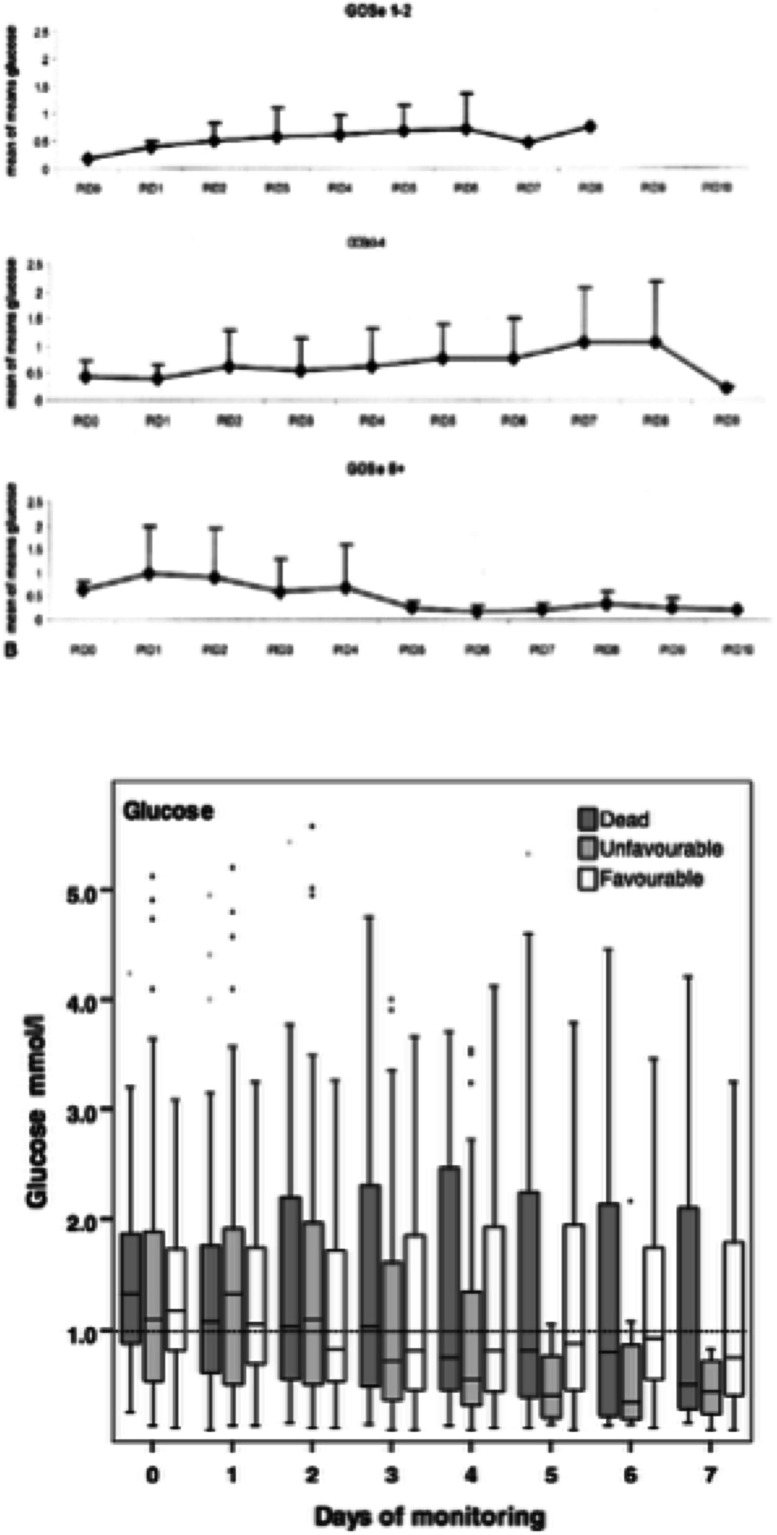
Pattern of declining microdialysate glucose after TBI: *Upper panel*: Graph showing initially ‘normal’ followed by declining mean microdialysate glucose concentrations after post-injury day 5 in those patients who went on to have a good outcome (Glasgow Outcome Scale extended (GOSe) 5 to 8). Figure originally published in the Journal of Cerebral Blood Flow & Metabolism (Vespa et al. 2003). *Lower panel*: Pooled microdialysis glucose concentrations averaged by day of monitoring and split by outcome categories demonstrating declining median concentrations over the course of a week of post-injury monitoring. Figure originally published in Brain (Timofeev et al. 2011a)

Patients with poorer outcomes after TBI have been observed to have lower average glucose concentrations for the monitoring period compared to those with better outcomes although the relationship is complex and high glucose concentrations are also associated with worse outcomes (Zauner et al. 1997; Vespa et al. 2003; Timofeev et al. 2011a; Yokobori et al. 2011). Vespa et al. (2003) identified three patterns of daily mean glucose concentrations in 30 patients with severe TBI. They found better outcomes when the glucose concentration was initially ‘normal’ and then later declined as opposed to initially low at the start of their monitoring (Fig. 3). They also found the lowest overall mean glucose in the patients that had the worse outcomes, although, no independent effect of low glucose was observed using a multivariate model with other known predictors. Similarly, Yokobori et al. (2011) found that glucose gradually declines until the fourth day after the onset of monitoring and then increases. The largest investigation of TBI patients to date found that the total averaged monitoring glucose concentration was a positive predictor of mortality in a multivariate model such that higher concentrations of glucose were associated with mortality (Fig. 3) (Timofeev et al. 2011a). Hence, the likelihood is that there is an optimum range for cerebral glucose with both low and high glucose having been associated with worse clinical outcomes.

Low brain glucose after TBI might be attributable to ischaemia, i.e. a deficiency in supply of oxygen, glucose and other blood-borne nutrients. Nowadays, with modern protocol-driven therapy, the incidence of gross ischaemia after TBI is low in neurocritical care patients. The injured brain may however be at risk from micro-vascular ischaemia, failure of vascular autoregulation, elevated intracranial pressure, and systemic problems that result in hypoxia and/or hypotension (Chesnut et al. 1993; Marmarou et al. 2009; Timofeev et al. 2011b; Newcombe et al. 2013). Because of the confounding effects of a reduced conscious level and sedative drugs after TBI ischaemic thresholds cannot simply be determined by CBF measurements. Instead an increase in the oxygen gradient across the brain is used to indicate a relative supply failure. This can be measured globally using jugular venous oxygen saturations and regionally using PET. Coles et al. (2004) used Oxygen-15 (^15^O) PET to measure oxygen extraction fraction (OEF) and to define ischaemic voxels. They based their OEF ischaemia threshold on a venous oxygen threshold of 3.5 ml/100 ml and estimated the volume of ischaemic brain in 15 TBI patients within 24 h of TBI. They found ischaemic volumes of between 1 and 16 % of the total brain volume, particularly in voxels adjacent to contusions or haematomas. Vespa et al. (2005), using similar methodology and the same OEF threshold in 19 patients found that the incidence of ischaemic voxels averaged only 0.14 ± 0.28 %, although patients underwent PET imaging at later time points; on average 60 ± 30 h from injury. In the same study, using a different threshold based on an OEF greater than 0.75 (a value based on previous ^15^O PET studies performed in patients with ischaemic stroke) they found the mean incidence of ischaemic voxels was 0.11 ± 0.18 % with a maximum of 1 % of voxels in a single patient. Furthermore, no significant correlation was found between microdialysis-measured brain glucose and PET measured CBF or OEF. Interestingly, in areas of brain deemed to be at risk of non-survival, defined as ^15^O PET-measured oxygen metabolism below a critical threshold, normobaric hyperoxia increases oxygen metabolism but does not have a consistent effect on microdialysis measures of metabolism (Nortje et al. 2008).

Although the incidence of frank ischaemia after TBI appears to be low, marked changes to CBF, induced by hyperventilation, or marked reductions in cerebral perfusion pressure (CPP) can affect brain glucose. Hutchinson et al. (2002) combined triple oxygen PET with microdialysis and found no significant correlation between CBF and brain microdialysate glucose in 17 patients with TBI. However, during periods of hyperventilation, which significantly increased the OEF, a significant reduction of brain glucose was observed. Hlatky et al. (2004) found that in a subset of 57 patients with TBI who experienced a marked reduction in CPP, due to high intracranial pressure (ICP) or systemic hypotension, there was a fall in brain glucose. This was paralleled by a fall in brain tissue oxygen reflecting the tissue ischaemia.

A reduction in brain glucose after TBI is partly a consequence of greater cellular uptake and utilisation, reflected by elevation of downstream metabolites. Pyruvate and lactate are both derived from glucose and so their extracellular concentrations partly reflect glycolytic activity. In turn, the relative proportions of lactate and pyruvate, the lactate/pyruvate ratio, reflects the degree that glycolytically produced pyruvate is subsequently metabolised oxidatively within the mitochondria via the TCA cycle or anaerobically in the cytosol to lactate. A greater lactate/pyruvate ratio indicates a greater proportion of glycolytic metabolism in relation to mitochondrial oxidative metabolism. Timofeev et al. (2011a) in the largest study to date found the most consistent trends of all the microdialysis parameters measured after TBI were for brain lactate and the lactate/pyruvate ratio both of which were lower on a daily basis in patients with favourable outcome as compared with those patients with a poor outcome. Moreover, the lactate/pyruvate ratio was found to be a significant positive predictor and pyruvate a significant negative predictor of mortality. The association of a higher lactate and higher lactate/pyruvate ratio with a poor outcome is a consistent finding across the TBI-microdialysis literature and is found in the most methodologically robust of studies that use patient-averaged data and multivariate statistical techniques (Timofeev et al. 2011a; Paraforou et al. 2011; Stein et al. 2012).

The association between high microdialysate lactate and poor outcome might seem, taken at face value, at odds with increasing evidence for lactate’s role as an energy substrate. However, this apparent contradiction may be explained by uncoupling of neuronal and glial metabolism. Animal studies have provided evidence for brain utilisation of intravenously administered 3-^13^C lactate via the TCA cycle (Tyson et al. 2003). In human brain, direct evidence for utilisation of lactate via the TCA cycle, in TBI patients, has been obtained in a cerebral microdialysis study in which 3-^13^C lactate was infused directly into brain via the microdialysis catheter (Gallagher et al. 2009). A recent intravenous lactate supplementation study in TBI patients revealed evidence for a beneficial effect judged by surrogate endpoints (Bouzat et al. 2014). Lactate has conventionally been regarded as a waste product of glucose metabolism, though a more recent idea is that neurons take up and metabolise lactate that has been generated by astrocytes. This has become known as the astrocyte-neuron lactate shuttle hypothesis (Pellerin and Magistretti 1994). Low extracellular lactate levels, associated with better outcomes (Timofeev et al. 2011a), might be because astrocyte-derived glycolytic lactate is efficiently taken up by neurons and utilised via the TCA cycle (Gallagher et al. 2009). Conversely, where neurons are too damaged to utilise the lactate produced from glucose by astrocytes, i.e. uncoupling of neuronal and glial metabolism, high extracellular levels of lactate would accumulate, explaining one potential mechanism behind the association between high extracellular lactate and poor outcome (Carpenter et al. 2014).

**Fig. 4:**
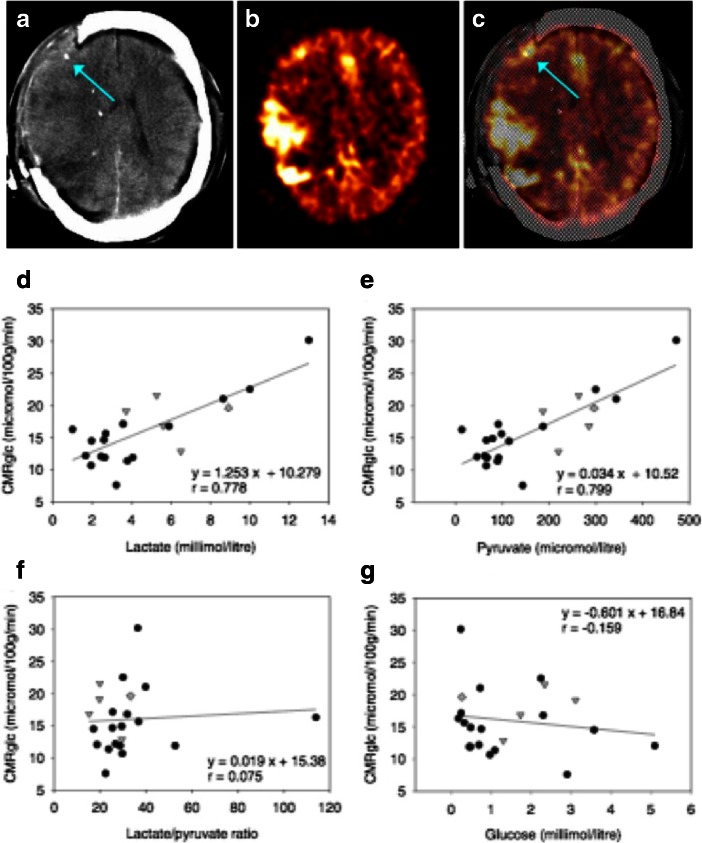
FDG-PET measurement of CMRglc and its relationship to brain microdialysate composition: **a**–**c** FDG-PET CMRglc map demonstrating relatively high FDG uptake at sites of injury, in contrast to less injured areas of the brain. **a** Computed tomography (CT) scan showing gold tip of microdialysis catheter (indicated by *arrow*). **b** Co-registered FDG-PET CMRglc map showing high FDG uptake at sites of injury. **c** Overlay of CT and co-registered CMRglc map, showing microdialysis catheter tip location (*arrow*). (**d**–**f** Graphs illustrating relationships by linear regression (for 22 ROIs in 17 TBI patients) between FDG-PET derived CMRglc and the microdialysis parameters measured during the scan (**d**) lactate, (**e**) pyruvate, (**f**) lactate/pyruvate (L/P) ratio, and (g) glucose. For the linear regressions in (**d**–**g**), corresponding values of p (ANOVA) are <0.0001, <0.0001, 0.74 and 0.48 respectively. Data-points from catheters at craniotomy sites (4 patients) are differentiated by *grey triangles*. Data-points from a second FDG-PET scan (one patient) are differentiated by *grey diamonds*. All other data-points are depicted as *black circles* (catheters inserted via cranial access device). Linear regressions presented on the graphs are for the entire (*combined black plus grey symbols*) dataset consisting of all 22 ROIs. Figure originally published in Acta Neurochirurgica (Hutchinson et al. 2009)

In support of brain glucose concentrations being influenced by uptake and cellular utilization, a combined ^18^F-fluoro-deoxyglucose-PET (FDG-PET) and cerebral microdialysis study in TBI patients found that the CMRglc measurements in a 2 cm region of interest around the microdialysis catheter tip showed significant positive correlations of CMRglc with lactate and pyruvate concentrations, no relationship between CMRglc and L/P ratio, and a weak inverse trend for CMRglc with glucose concentrations in the microdialysates (Fig. 4) (Hutchinson et al. 2009). Thus low concentration of brain extracellular glucose seems a consequence of increased substrate demand rather than inadequate substrate delivery. A caveat is that at low brain glucose concentrations there is a risk of underestimating lumped constant and hence of overestimating CMRglc in such situations. The main conclusion of the study is that in TBI brain an increase in glucose metabolism leads to increases in both lactate and pyruvate, as opposed to a shift towards anaerobic metabolism. For further discussion of PET in the context of cerebral metabolism see “Autoradiography and positron emission tomography” section below.

Brain glucose reflects systemic glucose levels, glycaemic control and the use of insulin in TBI patients. Several studies have explored the relationship between systemic glucose and brain microdialysis parameters in TBI patients and found that higher arterial glucose concentrations are associated with higher brain glucose concentrations although this relationship may be lost when the brain is injured (Vespa et al. 2006; Meierhans et al. 2010; Rostami and Bellander 2011). Furthermore, systemic glucose has an influence on other microdialysis parameters. Meierhans et al. (2010) observed that arterial glucose concentrations greater than 6 mmol/L were associated with both higher brain microdialysate glucose and glutamate concentration. Diaz-Parejo et al. (2003) observed that episodes of pronounced hyperglycaemia (greater than 15 mmol/L) were associated with greater brain lactate. The relationship between systemic glucose and cerebral metabolism has also been explored using AV measures of metabolism. Holbein et al. (2009) observed increased glucose uptake, decreased lactate export and reduced oxygen consumption by the brain at higher arterial glucose concentrations. The retrospective nature of these studies limits our ability to be clear about how changes in systemic glucose concentrations affect cerebral metabolism or to clearly define an optimal systemic glucose concentration for TBI patients. In a prospective study, Vespa et al. (2012) randomised 13 TBI patients using a within-subject crossover design to tight (80–110 mg/dL) versus loose (120–150 mg/dL) glycaemic control and assessed metabolism using both microdialysis and FDG-PET. They observed that microdialysate glucose, pyruvate and lactate were significantly lower, that more time was spent with a high lactate/pyruvate ratio (>28), and that FDG-PET indicated increased glucose metabolism (although in a heterogeneous manner depending on the patient and injury pattern) when tight glycaemic control was used. Hence, loose glycaemic control was interpreted as being preferable for cerebral metabolism.

In summary, microdialysis parameters can be difficult to interpret due to the various factors that influence the extracellular concentrations of metabolites. Trends of values and ratios, as opposed to single values, are more clinically useful in predicting clinical outcomes and for guiding treatments. Moreover, microdialysis is a focal technique limited to sampling a small area of brain. Data from microdialysis should be interpreted with this in mind; peri-contusional brain expresses a different metabolic profile to macroscopically normal brain. However, the relative ease of placement, safety and ability to follow trends in brain chemistry over extended periods of time has resulted in the adoption of microdialysis as a clinical monitoring tool in many neurocritical care units.

## Autoradiography and positron emission tomography

Autoradiography and PET imaging studies that use labelled 2-deoxy-D-glucose (2DG) as a tracer allow the in vivo delivery, uptake and initial metabolism of glucose to be assessed both globally and regionally in the brain. Demonstration of this technique by Sokoloff et al. (1977) provided some of the first experimental techniques for interrogating regional changes in cerebral metabolism in vivo. They used 2-deoxy-D-[^14^C]glucose as a tracer ([^14^C]DG), which is taken up from the circulation and phosphorylated by hexokinase in the first stage of glycolysis. [^14^C]DG, following phosphorylation, does not continue in the next and subsequent steps of glycolysis, essentially remaining trapped. Sokoloff et al. (1977) injected awake and anaesthetised rats with the tracer and used quantitative autoradiography of brain sections to calculate regional rates of glucose metabolism.

The principles of the autoradiography techniques developed by Sokoloff et al. (1977) have been adapted for use in humans with PET imaging. PET is a tomographic technique that involves measuring the accumulation of radiolabel in tissue following injection of labelled compound into the circulation. For the purposes of exploring regional glucose metabolism, the patient is injected with deoxy-glucose, labelled with the positron emitter ^18^F (FDG) (Phelps 1991). The emitted positrons are measured using a ring-shaped array of detectors (scintillator crystals and photomultipliers) and then algorithms used to reconstruct the location and local concentration of radioisotopes. Rate constants relating to the movement of tracer between the circulation, extracellular space and the intracellular compartment are used with the plasma glucose concentration and a ‘lumped constant’ to give a measure of the metabolic rate of glucose. The lumped constant (LC) of Sokoloff et al. (1977) accounts for the differences in transport and phosphorylation rates between D-glucose and 2DG. 2DG is more readily transported into the cell whereas glucose is more readily phosphorylated by hexokinase. However, there is no universal value for LC and values may differ between centres or even within-centre at different times. While many studies have assumed that the LC is uniform over the whole brain and in different states of disease or conscious level, this is not necessarily the case. Marklund et al. (2009) demonstrated regional differences in the uptake of ^14^C-glucose relative to 2DG in rats after experimental TBI, suggesting regional differences in LC, and Wu et al. (2004) showed that the global LC value was significantly lower in TBI patients compared with normal controls. Furthermore, various mathematical models have been used to calculate CMRglc from the PET data. These include the Huang autoradiographic model and dynamic methods such as Patlak graphical analysis and full kinetic modelling with 3 or 4 rate constants (termed 3 k or 4 k methods) (Huang et al. 1980; Patlak and Blasberg 1985) (Lammertsma et al. 1987). The different models apply different rate constants, which are either pre-defined (standard, as in the Huang method) or estimated from the FDG-PET data (as in the dynamic methods). Another approach is statistical parametric mapping to compare FDG uptake in different areas of the brain without calculating absolute CMRglc values (Zhang et al. 2010). Hence, it is not necessarily straightforward to compare FDG-PET quantitative results between studies performed in different centres, or within the same centre at different times.

Autoradiography and PET studies in animals have demonstrated a stereotyped acute period of high glucose metabolism followed by longer lasting metabolic depression. This acute period of hypermetabolism is observed in animal models for up to 6 h, and affects both hemispheres but more so on the ipsilateral hemisphere (Sunami et al. 1989). Following this acute period of hypermetabolism, a depression in glucose metabolic activity ensues that may last several weeks. This appears to be widespread throughout various regions of the brain and has been found to be associated with neurological outcome in clinical studies (Kato et al. 2007).

Most clinical reports demonstrate a depression of glucose metabolism following TBI when compared to control subjects with only discrete areas of supra-normal glucose metabolism, particularly in relation to mass lesions (Fig. 4). Bergsneider et al. (2000) observed that 20 of 26 (77 %) patients with a range of TBI severities within seven days post-injury demonstrated a CMRglc in cortical grey matter of the whole brain at least two standard deviations lower than a value for CMRglc derived from a historical control group (7.3 ± 1.2 mg/100 g/min). There was no clear correlation between the severity of injury and CMRglc values although patients that were on dual sedative drugs (morphine and a benzodiazepine) exhibited lower CMRglc values than those on a single sedative. Some peri-contusional cortical grey matter areas demonstrated markedly raised CMRglc values compared to the control value whereas others demonstrated depressed CMRglc. A later study from the same group, which used aged-matched controls and LC values calculated from a cohort of subjects who underwent additional arterio-venous 2DG gradient measurements, used to estimate a global LC value, found significantly lower CMRglc in the whole brain, striatum and thalamus in TBI patients compared to control subjects (Hattori et al. 2003). No differences were detected in the brainstem or cerebellum. Of note, whole brain CMRglc in control subjects in this study was 3.8 ± 0.7 mg/100 g/min, which was considerably lower than the value previously calculated from a historical cohort study.

Although glucose metabolism in TBI patients is found to be relatively depressed compared to awake and non-sedated control subjects, PET studies suggest that glucose metabolism in TBI is increased relative to the rate of oxygen utilisation. In a subset of six patients undergoing FDG-PET Bergsneider et al. (1997) used a combination of arterio-venous oxygen measurements and intravenous Xenon-133 CBF measurements to calculate both oxygen and glucose metabolism. In all six of these patients, who underwent FDG-PET imaging between days 2 and 8 post-injury, glucose metabolism was found to be disproportionately greater than oxygen metabolism, so termed ‘hyperglycolysis’. Wu et al. (2013) combined triple-oxygen (^15^O) PET, used to measure regional oxygen utilisation, with FDG-PET to explore the relationship between glucose and oxygen metabolism in contusional and peri-contusional regions in eight TBI patients. CMRglc in contusional and peri-contusional regions was depressed relative to CMRglc measured in the surrounding normal-appearing white matter of the same patient. Moreover, oxygen metabolism was even more depressed suggesting a shift in glucose metabolism from oxygen consuming to anaerobic metabolism.

The changes in glucose metabolic activity observed with deoxy-glucose studies relate to both the rate of glucose transport into cells and the rate of phosphorylation by hexokinase. Whether the changes in glucose metabolic activity that follow TBI are caused by changes in glucose transport, changes in hexokinase activity or are due to a combination of both changes in transport and hexokinase activity is not readily appreciated by autoradiography studies. Manipulating the mathematical models used in order to try and separate out these different aspects suggests that glucose transport activity is impaired in peri-contusional areas only whereas hexokinase activity is impaired more globally (Hattori et al. 2003; Wu et al. 2004).

Whether blood brain barrier (BBB) permeability impacts significantly on glucose metabolism in TBI patients is as yet unknown. A leaky BBB might conceivably allow more circulating glucose to enter the brain, while damaged cells might have less efficient specific transporters and/or exhibit a more glycolytic phenotype. In a microdialysis comparison of sites in TBI patients’ brains, glucose had a tendency to be lower in peri-lesional sites than in less-injured sites, while lactate/pyruvate ratio, lactate concentration and pyruvate concentration were all significantly higher in peri-lesional sites than in less-injured brain (Timofeev et al. 2011b). Although this was a large study (97 patients), a limitation of the statistical analysis was that catheters were not paired within-patient. A much smaller FDG-PET and microdialysis combined study included a subset of four patients with paired catheters (Hutchinson et al. 2009). In that study, the peri-lesional sites had higher pyruvate and higher regional CMRglc (together with a tendency towards higher lactate) than in the corresponding less-injured sites, while glucose and lactate/pyruvate ratio were not significantly different between sites, albeit in this very small subset of patients. More patients need to be studied for site-dependent differences, which may shed light on effects of BBB within the injured brain.

The infrastructure required to generate PET ligands as well as the ionising nature of the radiation limits their widespread use as a clinical tool. Furthermore, FDG-PET provides information on the uptake of glucose and on the first part of glycolysis but does not evaluate glucose metabolism beyond this.

## Carbon-13 labelling studies

**Fig. 5:**
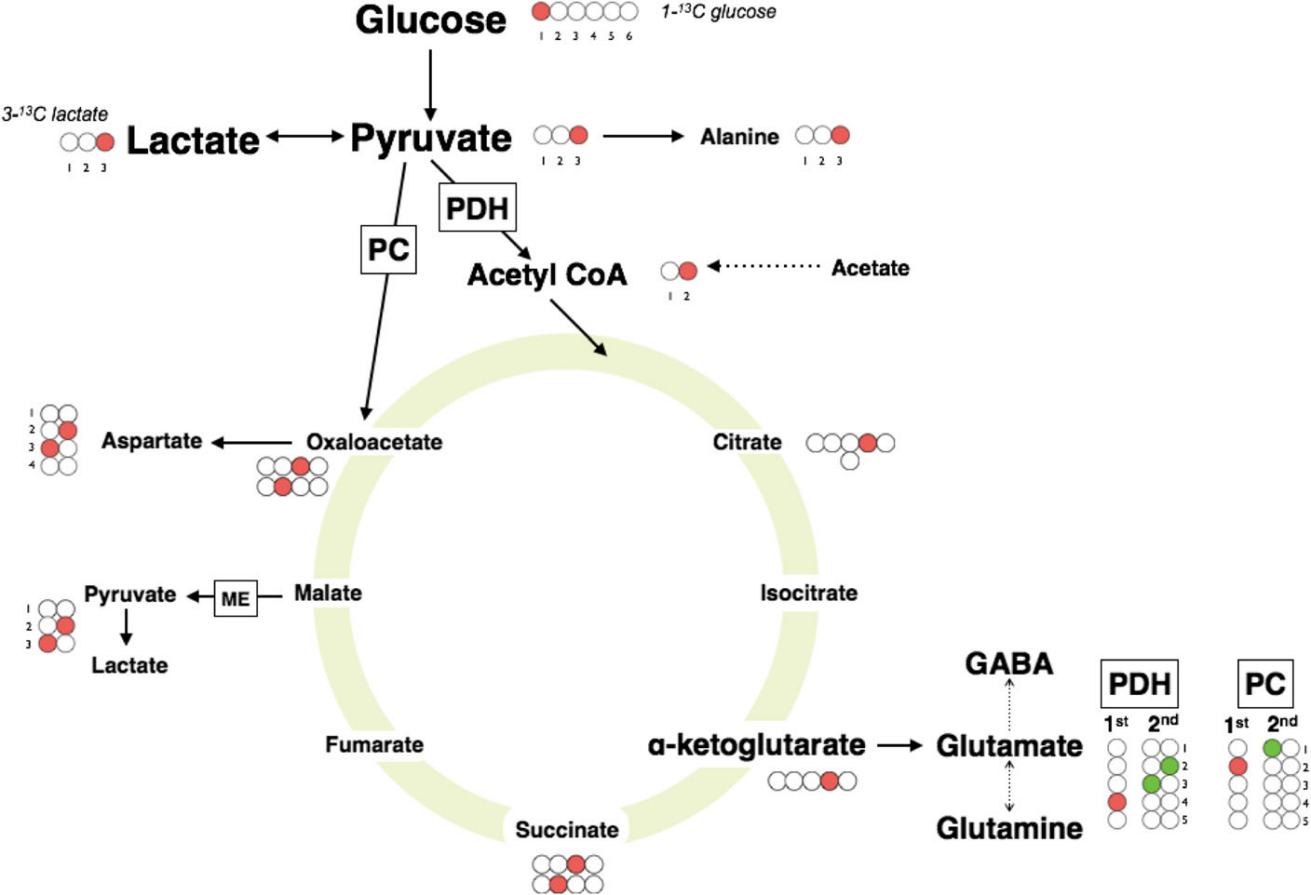
Carbon-13 labelling patterns after administration of 1-^13^C glucose: When the first carbon of glucose (C1) is ^13^C, the resulting pyruvate maintains the ^13^C as the third carbon (C3). Glycolysis converts one molecule of glucose into two molecules of pyruvate and so only one of the two 3-carbon chain length pyruvate molecules will contain ^13^C. Pyruvate can be converted to lactate, by the action of lactate dehydrogenase. Alternatively, through the action of pyruvate dehydrogenase on pyruvate, acetyl-CoA is produced containing just a 2-carbon backbone with one of the carbons (C1, the carboxylate carbon of pyruvate) lost as carbon dioxide. The 2 carbons of acetyl-CoA are then condensed with oxaloacetate and continue in the TCA cycle, ultimately emerging as amino acid spin-offs or as carbon dioxide. The main products identifiable with NMR (in animal brain tissue extracts ex vivo) or MRS (in human or animal brain in vivo) include glutamine, glutamate, GABA and aspartate. The C1 of a glucose molecule will become the C4 of glutamate and glutamine on the first turn of the TCA cycle. Subsequent turns of the TCA cycle will result in the ^13^C label shifting to the C3 or C2 of glutamate and glutamine. Hence, it is possible to determine, through the relative enrichment of the C4, C3, or C2 carbons, the proportion of glutamate or glutamine produced on the first turn of the TCA and subsequent cycles. In this way, ^13^C-NMR provides a convenient way to study the transformation of the carbon skeleton that takes place during the metabolism of glucose. The relative proportions of ^13^C incorporation at the different carbon positions of the relevant metabolites have been used in numerous studies to compare pathway activities. This schematic diagram is based on results obtained with singly labelled substrates, by Gallagher et al. (2009), Tyson et al. (2003) and Sampol et al. (2013) *Abbreviations*: *PDH* pyruvate dehydrogenase, *PC*, pyruvate carboxylase, *ME* malic enzyme

Applying a tracer to glucose that can then be identified in downstream metabolites derived from glucose allows the metabolic fate of glucose to be evaluated. Classically, this was achieved with radioisotope labelling using carbon-14 (^14^C). Due to the ionising nature of ^14^C these techniques are not readily applicable to patients. Moreover, most investigators using ^14^C-glucose have identified the labelled metabolites in extracted and purified brains. Carbon-13 (^13^C) is a naturally occurring stable isotope with magnetic properties that can be detected by nuclear magnetic resonance (NMR) spectroscopy and by gas chromatography–mass spectrometry (GC-MS). The ability of NMR to identify not only the metabolite but also the precise intra-molecular position(s) of ^13^C means that the relative activity of metabolic pathways can be determined from the particular labelling patterns observed in metabolites. See Fig. 5 for an example of labelling patterns observed after administration of 1-^13^C glucose and 3-^13^C lactate.

^13^C-labelled substrates have been delivered to the brain intravenously, or directly into the brain extracellular fluid using microdialysis. For an in-depth review see Carpenter et al. (2014). These studies have investigated whether the periods of ‘hyperglycolysis’ observed in studies using AV measurements or FDG-PET relate to actual increased glycolysis or are due to the metabolism of glucose by other alternative pathways, and have also investigated the metabolism of other substrates by the brain such as lactate and acetate (Gallagher et al. 2009; Carpenter et al. 2014).

^13^C-labelled glucose administered intravenously to CCI and FPI rat injury models of TBI, analysed by ^13^C NMR of brain tissue extracts, suggest that glucose is metabolised differently after TBI with up regulation of the pentose phosphate pathway (PPP) (Kaibara et al. 1999; Bartnik et al. 2005, 2007). The PPP is potentially an important reparative and protective pathway for the injured brain. It results in the production of ribose sugars, needed for DNA synthesis and repair, and the NADPH generated by the PPP also acts to maintain glutathione in a reduced state, which serves a vital antioxidant role (Herrero-Mendez et al. 2009). Dusick et al. (2007) used intravenously delivered 1,2-^13^C_2_ glucose and used GC-MS to measure the ^13^C-labelled products in arterial and jugular venous blood, in six TBI patients and six healthy controls. Their arterio-venous difference analysis of ^13^C-labelled lactate, differently labelled depending on whether it was derived by either glycolysis or PPP supported the preclinical finding of PPP up-regulation after TBI.

**Fig. 6:**
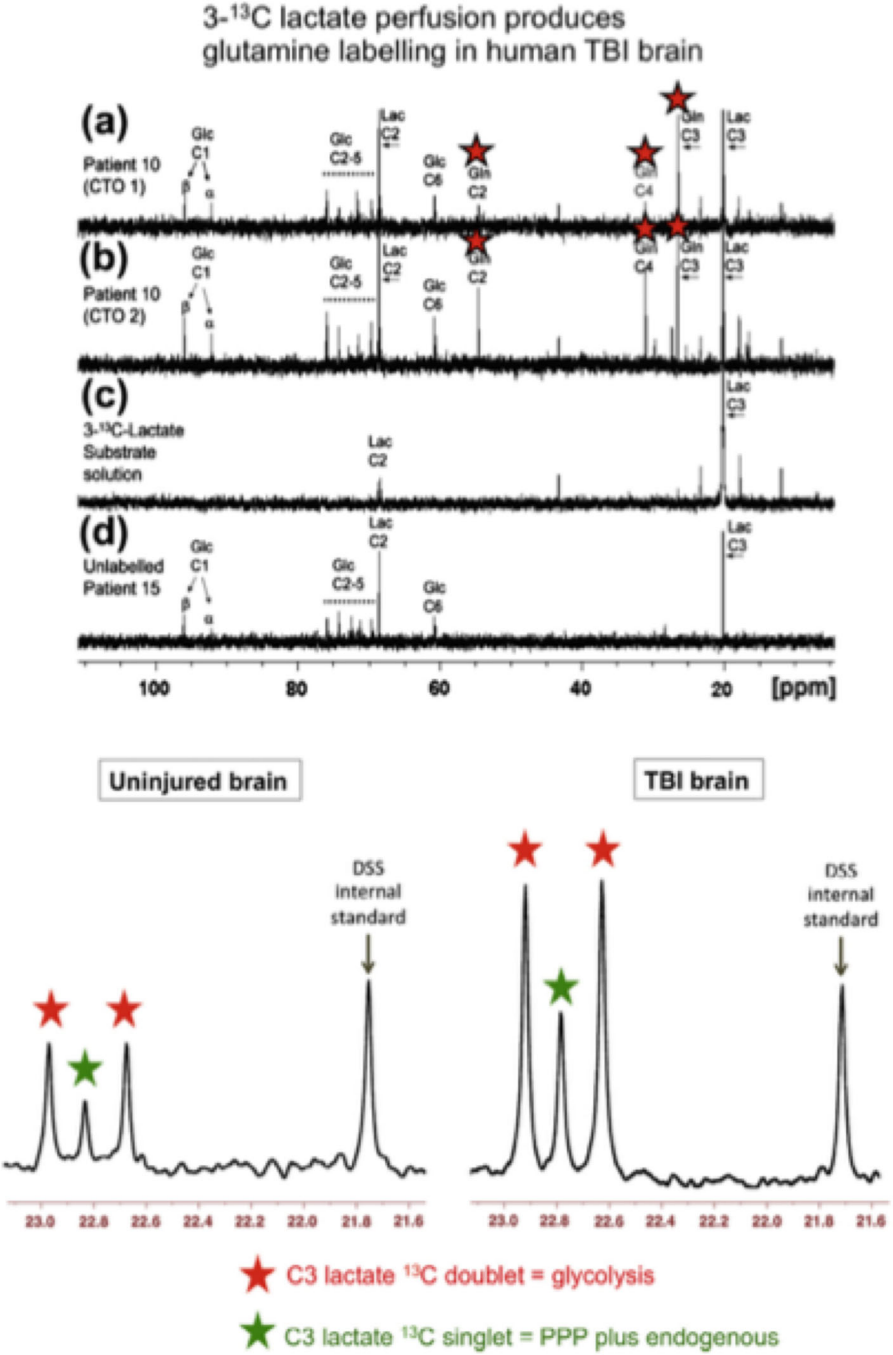
Illustrative ^13^C-NMR spectra achieved by ex vivo NMR of microdialysate after delivery of 3-^13^C lactate and 1,2-^13^C_2_ glucose to TBI patients: *Upper panel*: **a** and **b** Examples of ^13^C NMR spectra of brain microdialysates from a TBI patient, who received perfusion with 3-^13^C lactate (4 mM) by microdialysis catheters via a craniotomy (CTO); red stars indicate ^13^C signals for glutamine C4, C3 and C2 indicating metabolism via TCA cycle. **c** ^13^C NMR spectrum of the 3-^13^C lactate substrate solution prior to perfusing. **d** ^13^C NMR spectrum of brain microdialysate from an unlabelled patient. For further details, see Gallagher et al. (2009). Figure originally published in Brain (Gallagher et al. 2009). *Lower panel*: Examples of ^13^C NMR spectra of brain microdialysates from patients who received 1,2-^13^C_2_ glucose (4 mM) perfused via the microdialysis catheter. Uninjured brain is normal-appearing brain in a patient operated on for a benign tumour elsewhere in the brain. TBI brain is from a patient with a diffuse injury. The part of the spectrum illustrated in each case is for the C3 carbon of lactate. Also present in this part of the spectrum is one of the signals due to the internal standard DSS (2,2-dimethyl-2-silapentane-5-sulfonate sodium salt). The remainder of the spectrum (including the main DSS signal at 0 ppm) is not shown. The C3 doublet indicated by *red stars* represents lactate doubly labelled with ^13^C, produced by glycolysis; the C3 signal for ^13^C is split into 2 peaks by coupling to ^13^C also present at the neighbouring C2 position within the same molecule. The C3 singlet indicated by *green stars* represents lactate singly labelled with ^13^C, produced via the PPP. Figure originally published in European Journal of Pharmaceutical Sciences (Carpenter et al. 2014)

Examples of ^13^C NMR spectra of brain microdialysates are shown in Fig. 6. This includes spectra showing ^13^C signals for glutamine C4 after delivery of ^13^C-lactate to the brains of TBI patients indicating TCA cycle operation followed by conversion of glutamate to glutamine, which demonstrates that the injured brain is able to utilise lactate as a metabolic fuel (Gallagher et al. 2009).

The relatively novel combination of ^13^C-labelled substrate delivered by microdialysis with ex vivo NMR has some important advantages over other methods of studying metabolism in the clinical setting. Firstly, the technique has proved to be safe and easy to use in ventilated sedated TBI patients and does not require the transfer of the patient to a scanner (Gallagher et al. 2009). It is relatively inexpensive; only small amounts of ^13^C substrates (typically less than 1 g) are required to manufacture sufficient microdialysis perfusion fluid to study tens of patients. Furthermore, this technique allows the downstream metabolism of glucose to be evaluated beyond that previously afforded by arterio-venous studies and PET.

The intravenous delivery of exogenous ^13^C-labelled substrates can be combined with in vivo magnetic resonance spectroscopy (MRS) to investigate cerebral metabolism in humans, although this technique has not yet been applied to TBI patients. In vivo ^13^C-MRS, performed on a conventional MRI scanner with specialised coils, has been used to follow the rate of change of fractional enrichment of a metabolic product, performed in real time, after intravenous delivery of the substrate. In this way, infusing 1-^13^C glucose and measuring the rate of fractional enrichment for glutamate C4 has been used in metabolic modelling data analysis to calculate TCA cycle kinetics in healthy volunteers (Mason et al. 1995; Moreno et al. 2001; Gruetter et al. 2002; Rothman et al. 2011). The extension to TBI patients of such in vivo ^13^C MRS methodology will enable calculation of rates of metabolism beyond that previously afforded by FDG-PET, which only provides kinetic data on glucose uptake and phosphorylation, and will thus permit more in-depth evaluation of metabolism after TBI.

The TCA cycle is inherently mitochondrial, and the above ^13^C-labelling strategies with measurements by ex vivo high resolution NMR and in vivo MRS are thus relevant to mitochondrial activity in the human brain. In particular, microdialysis studies with ^13^C-labelled lactate and acetate have already demonstrated TCA cycle utilisation of these substrates in TBI patients’ brains (Gallagher et al. 2009). An important implication of these results is that mitochondria are evidently still active in injured brain, even in sites close to focal lesions. So although TBI brain is often regarded as “glycolytic”, and “mitochondrial dysfunction” invoked, the mitochondria are certainly functioning, although there is obviously much scope for debate about how healthy or otherwise they are. Also relevant in this context are results from a combined FDG-PET and microdialysis study in 17 TBI patients (Hutchinson et al. 2009). In that study, there was significant positive linear relationship between CMRglc and levels of lactate (*r* = 0.778, *p* < 0.0001) and pyruvate (*r* = 0.799, p < 0.0001), but not with the lactate/pyruvate ratio. This argued against a shift towards “anaerobic” metabolism at least in this cohort of patients. Taken together, the results imply that therapeutic strategies aimed at supporting or rescuing mitochondria may be helpful in TBI patients. Possibly relevant are experimental TBI studies in which Cyclosporin A, which inhibits opening of the mitochondrial permeability transition pore (MPTP), restored mitochondrial membrane potential, reduced tissue damage and improved neurological outcomes (Scheff and Sullivan 1999; Alessandri et al. 2002). Very recently, another experimental TBI study reported neuroprotective effects with improved behavioural outcomes for N-acetylcysteine amide (NACA), apparently due to maintenance of mitochondrial glutathione and mitochondrial bioenergetics, and to reduction in oxidative damage (Pandya et al. 2014). These or other mitochondrial support and rescue strategies may prove useful in future development of novel therapies for TBI. We have already mentioned (see above) the relevance of ^13^C labelling to address the TCA cycle in clinical studies of TBI patients. In addition, ^31^P in vivo MRS (discussed in “Proton & phosphorus magnetic resonance spectroscopy” section below) has the potential to be useful in future clinical trials of drugs directed at improving mitochondrial function by assessing the response of cellular ATP levels to treatment.

## Proton & phosphorus magnetic resonance spectroscopy

Magnetic resonance spectroscopy (MRS) is an in vivo technique carried out on suitably equipped MRI scanners. MRS was developed from the principles of NMR and can identify metabolites relevant to disordered energy metabolism in voxels and regions of interest (ROIs) defined on the MRI scan. The most commonly used nucleus is the proton (^1^H); however other nuclei that possess nuclei spin values of 1/2, and so in essence behave like miniature bar magnets, can also be used in MRS. ^1^H and phosphorus (^31^P) MRS have been used to interrogate energy metabolism after TBI. An important advantage of ^1^H and ^31^P MRS techniques is their ability to yield biochemical information non-invasively without the need for tracers. Different localisation techniques are used to produce spectra that represent either a broad region of the brain (e.g. a hemispherical ROI relative to the plane of a surface coil) or individual voxels (e.g. if a head-coil is used).

The sensitivity of ^1^H MRS is sufficient to allow the identification of N-acetylaspartate (NAA, typically the largest signal), choline-containing phospholipids, creatine and phosphocreatine, lactate, myo-inositol, glutamate, GABA, and lipids. The appearance of signals in ^1^H MRS is influenced by choice of echo time (TE). In particular, lactate signals are can appear positive, disappear or appear inverted depending on the duration of TE. Typically, in ^1^H MRS levels of lactate and other metabolites are expressed as ratios to creatine; absolute and accurate quantification of metabolite signals is more difficult (Marino et al. 2007, 2010).

The most relevant ^1^H MRS metabolite for demonstrating perturbed energy metabolism after TBI is lactate. Increases in lactate have been demonstrated after TBI particularly in children. In normal controls, a lactate peak is not readily detectable. In a study of 51 infants and children with TBI, 91 % of infants and 80 % of children with a poor outcome demonstrated a lactate MRS peak in the grey matter of the occipital lobe (Ashwal et al. 2000). A later study also showed that the presence of a lactate peak, this time with the MRS voxel located in the corpus callosum and frontal white matter, was predictive of a poor outcome in children (Aaen et al. 2010). The ability to detect lactate with MRS after TBI in children has not been consistently replicated in studies of adult patients. Several studies that demonstrate other markers of disordered biochemistry such as a reduced NAA/creatine ratio and an increased choline/creatine ratio did not find a detectable lactate signal in their patients (Cecil et al. 1998; Garnett et al. 2000). This may be due to the particular echo times used or the timing post-injury of the MRS scan. In support of this, MRS performed within 72 h of TBI was able to demonstrate a lactate peak in five of ten patients and this correlated with GCS at presentation and clinical outcome (Marino et al. 2007).

Adenosine triphosphate (ATP) an important end-point of cellular energy metabolism, and phosphocreatine (PCr), a readily mobilised store of high-energy phosphate, are compounds that can be measured in vivo using ^31^P MRS. When mitochondrial ATP synthesis is running normally, the PCr store is well stocked; when ATP synthesis is struggling to meet demand the PCr store runs down. ^31^P MRS has been used to determine the molar ratio of PCr to inorganic phosphate (PCr/Pi) and the PCr/ATP ratio as indicators of oxidative phosphorylation status and mitochondrial function (Eleff et al. 1984; Hetherington et al. 2001). Both PCr/Pi and PCr/ATP ratios are notionally easier to measure than absolute concentrations and have been measured in both chronic and acute TBI patients (Cadoux-Hudson et al. 1990; Garnett et al. 2001). Garnett et al. (2001) found increased PCr/Pi ratio and reduced Pi/ATP in predominantly normal appearing white matter of seven patients scanned between 2 and 21 days after severe TBI suggesting adequate mitochondrial energy metabolism. However, animal studies using ^31^P MRS after FPI or weight-drop model of diffuse injury reveal a decline in PCr/Pi suggestive of a failure of mitochondrial function (Vink et al. 1987; Heath and Vink 1995). More studies are needed to determine whether the differences between clinical and experimental findings are due to differences in pH, free intracellular magnesium concentrations, or cellularity, all of which might alter the measurement of phosphate compounds using ^31^P MRS.

## Summary and conclusions

Understanding cerebral energy metabolism is complicated by a series of specialisations that are unique to the brain. The brain exists in its own unique chemical environment, shielded by the blood brain barrier; it has high demands for energy and is vulnerable to reduced substrate provision. Unlike other organs, there are limited glycogen stores and the energy generating pathways are intrinsically linked to its main function of neurotransmission. Increasingly recognised are the functional differences in how the two main cell types, neurons and astrocytes, use glucose.

Changes to glucose metabolism that follow TBI appear to be largely independent of ischaemia. PET studies, both alone and in combination with microdialysis, suggest that ischaemia, when it occurs, is only detectable to any great extent in the first few hours after TBI or when CBF is challenged by hyperventilation or marked falls in CPP. More relevant is a change in the cellular utilisation of glucose with an increase in the proportion of glucose that is “anaerobically” metabolised. More studies are needed to determine whether this simply serves an increased requirement for a rapid supply of energy, maintaining the availability of high-energy phosphate compounds, or whether greater amounts of glucose are used for non-energy generating, reparative and biosynthetic roles such as the PPP. Along with a change in the metabolic role of glucose, lactate appears to become important as a metabolic substrate, imported from the circulation and oxidatively metabolised via the TCA cycle.

The methods discussed in this review are not yet employed widely in the clinical setting. They remain predominantly research tools but have the potential to become useful clinically. Choice of technique is inevitably dictated by what is available locally. In the present review we have gathered together findings from several technologies, and have discussed in each of the above sections what the particular technique is good for, and any limitations and caveats. A summary of methodology is also included (Table 1). A multi-technique approach is usually more powerful than a single methodology. Debate still exists on how best to integrate results from multiple techniques, including how to fit together “snapshot” techniques (such as scans) with data collected over an extended timescale of days (e.g. microdialysis), how to integrate global, regional and focal measures, and intracellular vs. extracellular information. Amalgamation of these diverse components may emerge from advances in data handling methods, including multivariate methods and machine learning technologies, when applied to the large databases that are growing in specialist centres and as a result of multicentre collaborations.

**Table 1:** Summary of methodology for measuring glucose metabolism in human brain

Technique	Extent of measurement	Timeframe (and frequency)	Invasive?	Examples in human brain
Arterio-venous difference	Global	Multi-day (sampling twice daily)	Yes (insertion of arterial line and jugular venous catheter)	Net changes (import or export) by brain for glucose and lactate (Jalloh et al. 2013)
Microdialysis	Focal	Multi-day (hourly vial changes)	Yes (insertion of catheter into brain)	Brain extracellular concentrations of small molecules (e.g. glucose, lactate, pyruvate, glutamate and glycerol) (Timofeev et al. 2011a).
PET	Global and regional	Usually single scan session (<1 h), sometimes repeated after a few days	Yes (i.v. injection of radioactivity with short half-life)	Regional cerebral metabolic rate of glucose (CMRglc) or oxygen (CMRO_2_)^a^ (Hutchinson et al. 2009; Hutchinson et al. 2002).
^1^H MRS	Regional and voxel	Usually single scan session (<1 h), sometimes repeated after a few days	No	Small molecules in brain tissue (e.g. NAA, creatine, choline, myo-inositol, glutamate and glutamine, GABA, lactate) (Marino et al. 2007)
^31^P MRS	Regional and voxel	Usually single scan session (<1 h), sometimes repeated after a few days	No	Phosphorus-containing small molecules in brain tissue (e.g. ATP, phosphocreatine, inorganic phosphate) and brain intracellular pH (Hamilton et al. 2006).
^13^C MRS	Regional and voxel	Usually single scan session (ca. 2 h)	Moderately (i.v. bolus and infusion of stable-isotope ^13^C-labelled substrate e.g. glucose)	^13^C-labelling in metabolites (glutamate and glutamine) for calculating TCA cycle rate, other ^13^C-labelled species also detectable (e.g. GABA, aspartate, NAA) (Rothman et al. 2011).
^13^C-labelled microdialysis	Focal	Typically 24 h (24 × 1 h vials pooled)	Yes (insertion of catheter into brain, perfused with solution of stable isotope ^13^C-labelled substrate e.g. glucose, lactate, or acetate)	^13^C- labelling patterns in metabolites (e.g. glutamine or lactate) diagnostic for biochemical pathways such as TCA cycle, glycolysis, PPP (Gallagher et al. 2009).

*Abbreviations: ATP* adenosine triphosphate, *CMR*_*glc*_ cerebral metabolic rate of glucose, *CMRO*_*2*_ cerebral metabolic rate of oxygen, *GABA* gamma-aminobutyric acid; *i.v.* intravenous, *MRS* magnetic resnance spectroscopy, *NAA* N-acetylaspartate, *PET* positron emission tomography, *PPP* pentose phosphate pathway

^a^CMRO_2_ measurement by PET additionally involves inhalation of radioactive (short half-life) gases

The precise biological questions to be addressed need to be appropriate to the techniques available. MRS and PET are suited to observing regional changes in metabolism and can demonstrate changes associated with structural lesions such as contusions. Currently, PET is limited by the availability of PET ligands, while MRS is limited by MR technology, improvements in which will lead to greater spatial resolution and the ability to interrogate all areas of the brain including deep structures such as the brain stem. This offers the potential to better characterise the injured brain, assisting with outcome prediction, and helping to direct clinical and rehabilitation management decisions. However, transferring patients from neurocritical care wards for PET or MRS scans is not a risk free undertaking, limiting the serial use of these investigations, so the time-variant aspect of such data is relatively unexplored and these scanning techniques are effectively snapshots for the duration of the scan. Moreover, only a few centres worldwide have scanners adjacent to neurocritical care wards and the necessary expertise for fully ventilated patients to routinely receive PET and MRI scans.

AV measurements and microdialysis are much more suited to following longitudinal changes. Moreover, the relative ease of obtaining data in the early stages following injury means they are particularly well placed to direct interventions and guide treatment. In particular, brain chemistry monitored by microdialysis has been demonstrated in large numbers of patients to relate statistically to clinical outcome and there is increasing evidence of how it is influenced by different therapeutic interventions. Hence, microdialysis is well placed to monitor glucose delivery to the brain, assist in the management of systemic glucose levels, and optimise CPP. Future clinical studies are needed to establish the role of microdialysis-directed interventions alongside ICP/CPP-guided management.

The current aims of TBI management focus on ICP/CPP-guided management to maintain adequate cerebral perfusion. The development of methods that sense metabolic changes means that we can tailor our interventions to maintain sufficient energy metabolism and ultimately limit the natural end-point of metabolic failure, cell death. The ability to monitor the changing metabolism of glucose after TBI raises interesting questions about the potential to manipulate these different pathways and reduce secondary injury. Multimodality monitoring, including microdialysis measures of brain chemistry, simultaneously with local PbtO_2_, together with ICP and derived pressure parameters such as CPP and PRx, is not only useful in standard neurocritical care to assist in patient management but is also a research approach that is being utilised to explore metabolic changes in the injured brain. Multimodality continuous monitoring is complemented by specialised scanning techniques (including PET, MRI and MRS), which provide a snapshot of the brain. The ability to understand and modulate glucose metabolism in the brain is likely to be a crucial factor in achieving the best clinical outcomes for TBI patients.
